# Probiotics, tetracycline fibres and chlorhexidine gel's effectiveness in treating chronic periodontitis as a supplement to scaling and root planning

**DOI:** 10.6026/973206300200933

**Published:** 2024-08-31

**Authors:** Abhigyan Manas, Jnana Ranjan Swain, Shivani Ambadas Kantale, Narayane R, Jeevan Prakash S, Pavithra Rangarajan Seshadri, Sabiha Kouser MS, Upasana Tyagi

**Affiliations:** 1 Department of Oral and Maxillofacial Surgery, Babu Banarsi Das College of Dental Sciences, Lucknow, UP, India; 2 Department of Pedodontics and Preventive Dentistry, Institute of Dental Sciences, Siksha O Anusandhan (Deemed to be University), Bubhaneswar, Odisha, India; 3 Department of Periodontology, Yogita Dental College and Hospital, Khed, Ratnagiri, Maharashtra, India; 4 Department of Periodontology, Indira Gandhi institute of Dental Sciences, Sri Balaji vidyapeeth, Puducherry, India; 5 Department of Periodontics, KGF Dental College, KGF-563115, Karnataka, India; 6 Departments of Periodontics, Ragas Dental College and Hospital, Uthandi, Chennai - 600119, Tamilnadu, India; 7 Periodontist, Implantologist and LASER Specialist, Senior Lecturer, Shubbaiah Institute of Dental Sciences, Shimoga, Karnataka, India; 8 Department of Public Health Dentistry, NIMS Dental College and Hospital, Jaipur, Rajasthan, India

**Keywords:** Chlorhexidine, Periodontitis, Probiotic, Tetracycline Fibers

## Abstract

A periodontal pocket can result into progressive loss of attachment. Many agents are tried to improve periodontal health. Hence this research was done to estimate the effectiveness of Tetracycline fibers; Probiotics and Chlorhexidine gel as a conjunction to scaling and root planning (SRP) in the treatment of chronic periodontitis. In all, 60 patients between the ages of 25 and 35 who had generalized chronic periodontitis and had a probing pocket depth (PPD) of at least 5 mm were enrolled for this study. Four groups of 15 patients each were created from the patients: Group 1: Tetracycline fibres after SRP, Group 2: Chlorhexidine gel, Group 3: Probiotic mouthwash and Group 4: Rinse with regular saline. At baseline, oral prophylaxis was administered to all groups. Gingival index (GI), Plaque index (PI), and probing depth (PD), among other clinical indicators, were evaluated at baseline, two weeks, and four weeks, respectively. All clinical findings, along withPlaque index (PI), Gingival index (GI), and Probing depth at various time intervals, decreased statistically significantly. Tetracycline fibres, Chlorhexidine gel, and probiotic mouthwash are all similarly effective for treating chronic periodontitis, but they are all more effective than SRP alone, according to an intergroup comparison. When it comes to SRP, local medication delivery methods are beneficial in treating periodontitis.

## Background:

The mouth is a portal or a doorway to many systemic health issues. The oral cavity acts as a holding area for a variety of microorganisms that can harm a person's overall health and compromise their immune system. At least 600 different bacterial species may exist in the mouth, and in some cases, more than 150 species may be found. Attached bacterial plaque on tooth surfaces may contain up to a billion bacteria, and maintaining proper dental hygiene is crucial for enhancing quality of life [1]. A set of inflammatory microbial-induced infections that affect the tissues that support the teeth are referred to as periodontal diseases. A periodontal pocket is created as a result of chronic periodontitis and a progressive loss of attachment. Additionally, many systemic disorders, including cardiovascular diseases, diabetes, and respiratory diseases, include periodontitis as an etiological factor or modifying factor [2]. However, due to the pathogenic bacteria's presence within gingival tissues or in other places that are inaccessible to periodontal instruments, mechanical therapy alone may fall short of completely eliminating the germs. As a result, the use of various antimicrobial agents began to gain popularity as chemical tools to make up for technical shortcomings and stop early recolonization of microbes, thereby make sure the best possibility for clinical benefits [3^3^]. One of the chemical assistance for periodontal therapy is probiotic. Probiotics are defined as "live microorganisms that, when administered in adequate amounts, confer health benefits on the host" [4]. They replenish the good bacteria, which can aid in the eradication of pathogenic bacteria and the prevention of infection [5]. Despite its numerous negative effects, chlorhexidine is a chemical agent that is regarded as the gold standard in dentistry for the avoidance of dental plaque [6]. Tetracycline fibres have the ability to bind to the surface of the roots and can release the active form of the drug over an extended period of time. It lessens the co-aggregation and adhesion abilities of some bacteria linked to illness, such as *P. gingivalis* and *P. intermedia* [7]. These medications have been used either alone or in conjunction with SRP to stop the spread of periodontal disease. As a result, the current study compared the effectiveness of three chemical agents, namely probiotics, tetracycline fibres, and chlorhexidine gel, when used in conjunction with SRP to treat chronic periodontitis. The purpose of this research was to estimate the effectiveness of Tetracycline fibers; Probiotics and Chlorhexidine gel as a conjunction to scaling and root planning (SRP) in the treatment of chronic periodontitis.

## Materials & Methods:

After receiving institutional ethics committee approval, 60 systemically healthy patients between the ages of 25 and 35 were recruited from the outpatient Department of Periodontology (25 females and 20 males). Before the research began, informed permission was taken from all subjects. Ethical approval was obtained from institutional ethics committee. This is a cross sectional observational study.The inclusion criterion for this research were; patients with generalized interproximal attachment loss, and generalized Chronic periodontitisdefined by probing depth ≥ 4 mm to ≤ 7mm and Patients having more than 20 teeth i.e. minimum 5 teeth per quadrant. The exclusion criteria were; Patients having systemic diseases, Pregnant/lactating females, History of anti-biotic therapy, History of any deleterious habit like smoking or Para-functional habit like mouth breathing, Patients with orthodontic/ prosthodontic appliances Patients were arbitrarily categorized into four groups; Group 1- Tetracycline fibers placed in the periodontal pocket surrounding the tooth after SRP, Group 2-Chlorhexidinegelwhich was placed in the periodontal pocket after SRP, Group 3: rinse with Probiotic mouthwash after SRPundiluted for 1 min twice daily, 30 min after brushing and Group 4 were advised to rinse with Normal saline after SRP undiluted for 1 min twice daily, 30 min after brushing.Gingival index (GI), Plaque index (PI), and probing depth (PD), among other clinical indicators, were assessed on day 0 (Baseline), then at intervals of two and four weeks. All groups had baseline full mouth ultrasonic scaling.

## Statistical Analysis:

The obtained data was evaluated with SPSS software version 23.0.UsingTukey's tests, ANOVA, and Friedman tests with P<0.05.

## Results:

Intragroup comparison in Tetracycline group shows statistically considerable decrease in PI, GI and PD from Baseline to 15 days, Baseline to 1monthand 15 days to 1 month (Table 1). Table 2 shows intragroup comparison in Chlorhexidine gel group at various time intervals. Likewise Tetracycline fibres group, statistically considerable decrease was found in PI, GI and PD from Baseline to 15 days, Baseline to 4 weeks and 2 weeks to 4 weeks. Table 3 shows intragroup comparison in Probiotic mouthwash group at different time intervals. A statistically considerable decrease was observed in PI, GI and PD from Baseline to 15 days, Baseline to 4 weeks and 2 weeks to 4 weeks, whereas statistically non-significant reduction was observed from 2 weeks to 4 weeks for all clinical parameters.

Table 4 shows intergroup comparison of Plaque Index. It indicates statistically considerable dissimilarity in Tetracycline fibres and Probiotic mouthwash, Chlorhexidine gel and Probiotic mouthwash, Tetracycline fibres and Control group, Chlorhexidine gel and Control group, and Probiotic mouthwash and Control group at all-time intervals whereas statistically non-considerable variation was observed in Tetracycline Fibers and Chlorhexidine gel group. Table 5 shows intergroup association of Gingival Index. It reveals statistically considerable variation in Tetracycline fibres and Probiotic mouthwash, Chlorhexidine gel and Probiotic mouthwash, Tetracycline fibres and Control group, Chlorhexidine gel and Control group, and Probiotic mouthwash and Control group whereas statistically non-significant difference was observed in Tetracycline Fibers and Chlorhexidine gel at all-time intervals.

Table 6 shows intergroup comparison of Probing depth. It reveals statistically significant difference in Tetracycline fibres and Chlorhexidine gel, Tetracycline fibres and Probiotic mouthwash, Tetracycline fibres and Control group, Chlorhexidine gel and Control group, and Probiotic mouthwash and Control group whereas statistically non-significant difference was observed in Chlorhexidine gel and Probiotic mouthwash at all-time intervals.

## Discussion:

In addition to mechanical control, chemical agents are used regularly in clinical practise. They are meant to support mechanical plaque control, not to replace it [5]. These antibacterial substances work by broadly lowering the populations of both beneficial and dangerous oral microorganisms. Probiotics, on the other hand, were created using naturally occurring beneficial bacteria to support a balanced population of oral microbes [8, 9]. Scaling and root planning is one of the non-surgical mechanical mode to remove etiological factors contributing in the periodontal infection. SRP has been the gold standard for periodontal therapy when combined with good dental hygiene practices [10]. Probiotic technology promises a new way to maintaining oral health by utilising beneficial bacteria that are naturally present in healthy oral cavity to create a natural defence against those germs regarded to be damaging to teeth and gums [11, 12]. Chlorhexidine gel has the antibacterial property which acts by non-specifically lowering the number of both harmful and friendly oral bacteria. Clinical data were recorded in the current investigation within a month because it is alleged that after 3 to 6 weeks of SRP, the bacterial flora will resume its pre-treatment patterns. It is in agreement to the fin dings of Harpreet *et al.* study [8].

## Intragroup comparison:

## Tetracycline fibres:

Decrease in PI, GI and PD score was found to be statistically considerable in Tetracycline fibres group. The reason for this reduction could be because of chemical control by the Tetracycline fibres placed subgingivally which could also resulted in inhibitory effect on supra gingival plaque. This effect helped in the lessening of accumulation of plaque resulting onto gingival inflammation and formation of periodontal pocket. It is in accordance to the observations made by Sharma *et al.* who observed reduction in plaque index in patients treated with tetracycline fibres[13]. The reduction in PD was supported by a study conducted by Friesen *et al.* Perinetti *et al.*[14, 15].

## Chlorhexidine gel:

Diminution in PI, GI and PD score was observed to be statistically considerable in Chlorhexidine gel group. The probable reason for his reduction could be antiplaque and antibacterial property of chlorhexidine, which may have leaked out from the pockets and better oral hygiene practiced by the patients[16]. It is in accordance to the research done by Vinholis *et al.*[17], Vaidya *et al.*[18]. They also observed reduction in the PD after administration of chlorhexidine gel in pocket depths. The decrease in the score of all the three clinical parameters can relate to the bactericidal concentrations of the Chlorhexidine gel. These results of the current research were in disparity to the study conducted by Azmak *et al.* [19].

## Probiotic mouthwash group:

Reduction in PI, GI and PD score was found to be statistically considerable in Probiotic mouthwash group. It is in harmony to the research conducted by Shiva *et al.*[10]. In the oral cavity, probiotics reduce pH, which prevents periodontopathogens from forming calculus and plaque, which is the main etiological factor 10-shiva.It is also in conjugation with study conducted by Teughels *et al.* [20]. They found significant reduction in *P.gingivalis* levels, more pocket depth reduction and attachment gain in SRP + Probiotic mouthwash group [2].In another study by Nada *et al.*it was found that administration of beneficial bacteria in the form of probiotics can be a helpful option in the treatment of periodontitis [21].

## Control Group:

In this group all the clinical parameters i.e. PI, GI and PD showed reduce score but statistically it was observed to be non-significant. The probable reason could be that, in control group only SRP was done which was not alone effective and efficient for the management of periodontitis.

## Intergroup comparisons:

A statistically non-significant variation was found in Tetracycline fibres and Chlorhexidine gel for PI and GI whereas statistically significant difference was observed for PD [21]. The findings are in compliance with Unsal *et al.*, who assessed the outcome of 10% tetracycline paste and 2% chlorhexidine gel along with the SRP [22]. It is also supported by another study conducted by Harpreet *et al.* [8] who also observed the findings in their study. Comparable interpretations were made by Banodkar and Rao [23]. Tetracycline has bactericidal and bacteriostatic effects on periodontal bacteria, as well as the ability to enhance fibroblast attachment to root surface by adsorption to dental surfaces. These findings were in distinguished with the observation of Wilson et al study [24]. The outcomes are consistent with those of Goodson *et al.* [25], and Newman *et al.* [26] who noted a larger decrease in probing pocket depth in the group receiving tetracycline plus SRP. In contrast, to study done by Munishwar *et al.* [27] observed better clinical results with Tetracycline fibres and SRP as compared to chlorhexidine chips. A statistically significant difference was observed in Tetracycline fibres and Probiotic mouthwash, Chlorhexidine Gel and Probiotic mouthwash for all the clinical parameters except for PD, in which Chlorhexidine Gel and Probiotic mouthwash showed statistically non-significant difference. These findings are supported by study conducted by Sharma *et al.* [13] who also observed reduction in plaque index in patients treated with tetracycline fibres.

## Limitations:

Smaller sample size further long term researches are required to assess the efficacy of these materials on different population groups.

## Conclusion:

It can be observed from this research that scaling and root planning is not very effective for the management of periodontal pockets alone. All these chemical agents along with SRP have shown better results. The patients tolerated the entire materials well and they had good biological acceptability. Thus, combined with SRP, all three chemical agents were both secure and effective in the treatment of periodontal disorders.

## Financial support and sponsorship:

Nil

## Competing Interests:

None

## Figures and Tables

**Table 1:** Comparing the intra groups of the Tetracycline Group at different time points

**Tetracycline Fibers**	**Baseline-2 weeks**	**Baseline-4 weeks**	**2 weeks-4 weeks**
PI mean reduction	0.42 ± 0.51	0.32 ± 0.57	0.35 ± 0.37
p-value	<0.001	<0.001	<0.001
Mean reduction in GI	0.38 ± 0.64	0.31± 0.70	0.22 ± 0. 82
p-value	<0.001	<0.001	<0.001
Mean reduction in PD	0.24 ±0.52	0.22 ±0.10	0.20 ±0.24
p-value	<0.001	<0.001	<0.001

**Table 2:** Comparing intra groups in the Chlorhexidine gel Group at different times

**Chlorhexidine Gel**	**Baseline-2 weeks**	**Baseline-4 weeks**	**2 weeks-4 weeks**
PI mean reduction	0.75±0.45	0.65±0.53	0.50±0.65
p-value	<0.001	<0.001	<0.001
Mean reduction in GI	0.55 ± 0.40	0.53 ± 0.72	0.47 ± 0.61
p-value	<0.001	<0.001	<0.001
Mean reduction in PD	0.64 ±0.67	0.48 ±0.35	0.40 ±0.35
p-value	<0.001	<0.001	<0.001

**Table 3:** Intragroup comparison in the Probiotic mouthwash group at different time points

**Probiotic mouthwash**	**Baseline-2 weeks**	**Baseline-4 weeks**	**2 weeks-4 weeks**
PI mean reduction	0.60 ± 0.52	0.53 ± 0.49	0.45 ± 0.22
p-value	<0.001	<0.001	0.003
Mean reduction in GI	0.52 ± 1.07	0.49 ± 0.098	0.38 ± 0.81
p-value	<0.001	<0.001	0.012
Mean reduction in PD	0.44 ± 0.32	0.43 ± 0.26	0.32 ± 0.23
p-value	<0.001	<0.001	0.021

**Table 4:** Plaque Index intergroup comparison at various time intervals

**Plaque Index**	**Baseline**	**2 weeks**	**4 weeks**
Tetracycline Fibers VS Chlorhexidine gel	165	147	154.5
p-value	0.28	0.83	0.85
Tetracycline Fibers VS Probiotic mouthwash	134.8	126	132
p-value	<0.001	<0.001	<0.001
Chlorhexidine gel VS Probiotic mouthwash	142	123	128
p-value	<0.001	<0.001	<0.001
Tetracycline Fibers VS Control group	155	132	123
p-value	<0.001	<0.001	<0.001
Chlorhexidine gel VS Control group	143	132	137
p-value	<0.001	<0.001	<0.001
Probiotic mouthwash VS Control group	157	162	153
p-value	<0.001	<0.001	<0.001

**Table 5:** Intergroup comparison of Gingival Index at different time intervals

**Plaque Index**	**Baseline**	**2 weeks**	**4 weeks**
Tetracycline Fibers VS Chlorhexidine gel	173	152	164
p-value	0.36	0.57	0.82
Tetracycline Fibers VS Probiotic mouthwash	165	155	148
p-value	<0.001	<0.001	<0.001
Chlorhexidine gel VS Probiotic mouthwash	152	140	155
p-value	<0.001	<0.001	<0.001
Tetracycline Fibers VS Control group	148	144	132
p-value	<0.001	<0.001	<0.001
Chlorhexidine gel VS Control group	142	138	140
p-value	<0.001	<0.001	<0.001
Probiotic mouthwash VS Control group	152	145	148
p-value	<0.001	<0.001	<0.001

**Table 6:** Intergroup comparison of Probing depth at different time intervals

**Plaque Index**	**Baseline**	**2 weeks**	**4 weeks**
Tetracycline Fibers VS Chlorhexidine gel	162	141	153
p-value	<0.001	<0.001	<0.001
Tetracycline Fibers VS Probiotic mouthwash	124	118	127
p-value	<0.001	<0.001	<0.001
Chlorhexidine gel VS Probiotic mouthwash	148	140	162
p-value	0.009	0.082	0.217
Tetracycline Fibers VS Control group	142	139	137
p-value	<0.001	<0.001	<0.001
Chlorhexidine gel VS Control group	158	146	148
p-value	<0.001	<0.001	<0.001
Probiotic mouthwash VS Control group	165	153	142
p-value	<0.001	<0.001	<0.001
